# Correction to Vaterite Dissolution: Mechanism and Kinetics

**DOI:** 10.1021/acs.jpcc.4c06304

**Published:** 2024-09-30

**Authors:** Morgan P. Milner, Minjun Yang, Richard G. Compton

Corrections:

- The labels V and C in Figure S6 of the Supporting Information should be interchanged.
- The reported particle concentrations for the dissolution study are a factor of 10 too large throughout; i.e., a range of 4 μg mL^–1^ to 45 μg mL^–1^ was studied rather than 40 μg mL^–1^ to 450 μg mL^–1^.

The conclusions, both qualitative and quantitative, in respect of the vaterite dissolution kinetics and mechanism are unchanged.

## Supporting Information Available

Corrected Figure S6: XRD of vaterite particles synthesised with a 15-minute growth period (PDF)

