# Supplementary material for: Correction to Vaterite Dissolution: Mechanism and Kinetics
Source: J Phys Chem C Nanomater Interfaces. 2024 Sep 30;128(40):17196. doi: 10.1021/acs.jpcc.4c06304 (PMC11472371; doi:10.1021/acs.jpcc.4c06304)
Supplement: Supplementary file 1 — jp4c06304_si_001.pdf [file jp4c06304_si_001.pdf]

Correction to Vaterite Dissolution: Mechanism and Kinetics

Authors: Morgan P. Milner, Minjun Yang, Richard G. Compton*

Milner, M. P.; Yang, M.; Compton, R. G. Vaterite Dissolution: Mechanism and Kinetics. *The Journal of Physical Chemistry C* **2024**, *128* (25), 10388–10396. https://doi.org/10.1021/acs.jpcc.4c02074.

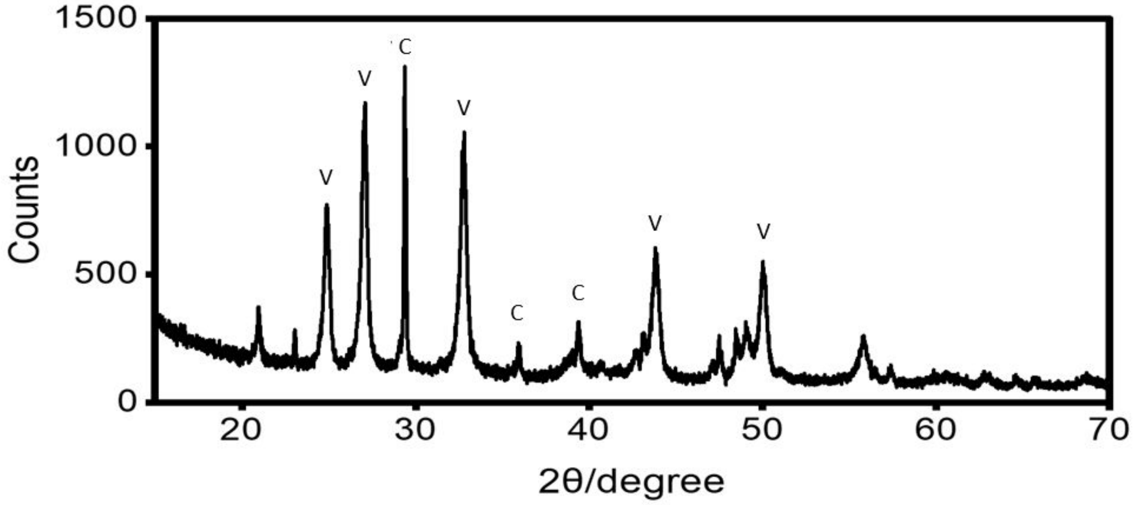

Figure S6. XRD of vaterite particles synthesised with 15-minute growth period, distinctive peaks associated with calcite and vaterite clearly visible. Clear, non-overlapping peaks are labelled as V for vaterite and C for calcite in accordance with literature.
